# On the identification of power-law creep parameters from conical indentation

**DOI:** 10.1098/rspa.2021.0233

**Published:** 2021-08

**Authors:** Yupeng Zhang, Alan Needleman

**Affiliations:** Department of Materials Science and Engineering, Texas A&M University, College Station, TX, USA

**Keywords:** indentation, power-law creep, material properties, Bayesian statistics

## Abstract

Load and hold conical indentation responses calculated for materials having creep stress exponents of 1.15, 3.59 and 6.60 are regarded as input ‘experimental’ responses. A Bayesian-type statistical approach (Zhang *et al.* 2019 *J. Appl. Mech.* **86**, 011002 (doi:10.1115/1.4041352)) is used to infer power-law creep parameters, the creep exponent and the associated pre-exponential factor, from noise-free as well as noise-contaminated indentation data. A database for the Bayesian-type analysis is created using finite-element calculations for a coarse set of parameter values with interpolation used to create the refined database used for parameter identification. Uniaxial creep and stress relaxation responses using the identified creep parameters provide a very good approximation to those of the ‘experimental’ materials with stress exponents of 1.15 and 3.59. The sensitivity to noise increases with increasing stress exponent. The uniaxial creep response is more sensitive to the accuracy of the predictions than the uniaxial stress relaxation response. Good agreement with the indentation response does not guarantee good agreement with the uniaxial response. If the noise level is sufficiently small, the model of Bower *et al.* (1993 *Proc. R. Soc. Lond. A* **441**, 97–124 ()) provides a good fit to the ‘experimental’ data for all values of creep stress exponent considered, while the model of Ginder *et al.* (2018 *J. Mech. Phys. Solids* **112**, 552–562 ()) provides a good fit for a creep stress exponent of 1.15.

## 1. Introduction

The serviceability and reliability of a variety of engineering components, as for example, in turbines used for electricity generation and in vehicle and aeroplane engines, are limited by continuing deformation at relatively low stress levels, i.e. creep. Instrumented indentation is attractive for identifying creep properties as it is non-destructive, requires a relatively small specimen, and has been used for the identification of mechanical properties of a broad range of materials. However, indentation involves a complex deformation field, and extracting material properties from experimentally measured indentation quantities can be complex and non-unique.

The creep deformation of polycrystalline structural metals often can be characterized appropriately by an isotropic power-law creep constitutive relation and there is a large literature on modelling the indentation response of power-law creeping materials using analytical methods, numerical methods or a combination of these (e.g. [1–6]). In particular, studies have been carried out using such analyses to extract power-law creep parameters from indentation responses, including, for example, [7–14]. Specifically, in [2,10–12] experimental creep indentation data were related to uniaxial power-law creep properties using analytical results from Bower *et al.* [1] and from the expanding cavity model of Ginder *et al.* [2].

Here, the Bayesian statistics-based approach of Zhang *et al.* [15] is used to extract power-law creep parameters from the indentation depth versus time response and the residual surface profile. Finite-element solutions for three materials with very different power-law creep properties are considered to be the ‘experimental’ responses. The power-law creep parameters identified via indentation, using noise-free as well as noise-contaminated data, are compared with the corresponding uniaxial creep and stress relaxation responses of the input ‘experimental’ materials.

The questions addressed include:

(i) Can very different power-law creep parameters give nearly the same responses in load and hold indentation creep? There are sets of rate-independent plastic material parameters that have indistinguishable force versus depth responses in conical indentation but very different uniaxial responses [15–17].
(ii) Does using the residual surface profile in addition to or instead of the indentation depth versus time data improve the quality of the prediction?
(iii) How sensitive is the predicted creep response to noise in the ‘experimental’ indentation data?
(iv) How do the power-law creep properties obtained using the analytical steady-state creep results of Bower *et al.* [1] and Ginder *et al.* [2] compare with those predicted from the Bayesian-type statistical approach?

## 2. Problem formulation

**Figure 1.**
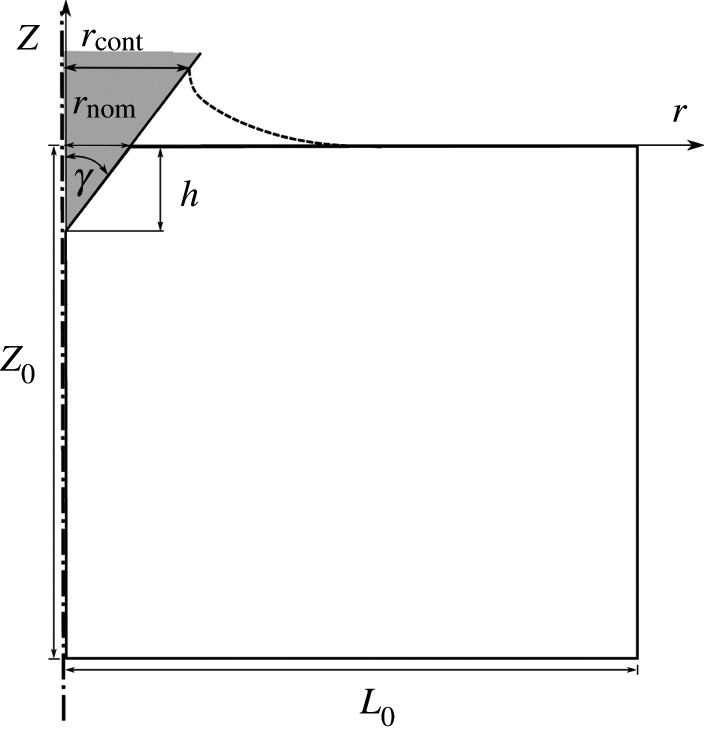
Sketch of the indentation configuration analysed with h the indentation depth magnitude, $r_{\mathrm{cont}}$ the actual contact radius and $r_{\mathrm{nom}}$ the nominal contact radius.

Indentation into an isotropic elastic power-law creep solid by a conical indenter is modelled as sketched in figure 1. Quasi-static loading conditions are presumed. The dimensions of the region analysed are taken to be large enough to approximate indentation into a half-space and the deformations are restricted to be axisymmetric.

Calculations are carried out for an indenter angle $\gamma =70.3^{\circ }$, which is the equivalent conical indenter angle to a Berkovich indenter assuming the same projected area on contact at a given indentation depth [18]. The indentation force in the z-direction is a prescribed function of time, the nominal indentation depth magnitude is denoted by h and the corresponding nominal contact radius is $r_{\mathrm{nom}}=h\tan\gamma$ (figure 1).

The calculations are carried out using a quasi-static Lagrangian implementation in the commercial finite-element program ABAQUS [19] standard. Elastic deformations are presumed small but finite creep strains are accounted for.

### (a) Initial/boundary value problem

The magnitude of the indentation force in the z-direction, $P_{z}$, is a prescribed function of time, f(t), so that

2.1 $$P_{z}=\int_{S_{\mathrm{contact}}}T_{z}\;ds=f(t),$$

where $T_{z}$ is z-component of the traction vector, T, on the contact surface $S_{\mathrm{contact}}$.

As described in the ABAQUS [19] manual, the remaining conditions imposed on $S_{\mathrm{contact}}$ are

2.2 $$T_{t}=\mu T_{n}\;\mathrm{and}\;\min[\int_{S_{\mathrm{contact}}}{\left(\Delta {\dot{u}}_{n}\right)}^{2}\;ds].$$

Here, $T_{t}$ and $T_{n}$ are the components of T in the directions tangent and normal, respectively, to the indenter, and $\Delta {\dot{u}}_{n}$ is the difference in displacement rate components normal to the indenter, with $\left(\dot{}\right)$ denoting the time derivative.

The coefficient of friction is taken to be μ=0.4, leading to very little slip along the contact surface and the contact boundary conditions in normal direction of equation (2.2) are termed ‘hard contact’ in the ABAQUS [19] standard manual.

With r and z denoting the positions of material points in the initial configuration, the remaining boundary conditions are

2.3a $$\begin{array}{rl} & T_{r}=T_{z}=0\;\mathrm{on}\;r=L_{0}\;\mathrm{and}\;z=0,\;S\neq S_{\mathrm{contact}}, \end{array}$$

2.3b $$\begin{array}{rl} & {\dot{u}}_{r}=0,\;T_{z}=0\;\mathrm{on}\;r=0, \end{array}$$

2.3c $$\begin{array}{rl} & {\dot{u}}_{z}=0,\;T_{r}=0\;\mathrm{on}\;z=-Z_{0}. \end{array}$$

### (b) Constitutive relation

The elastic-creep constitutive relation of ABAQUS [19] standard is used so that the (small) elastic strain–stress relation has the form

2.4 $$\epsilon^{e}=\frac{\left(1+\nu \right)}{E}\tau -\frac{\nu }{E}\mathrm{tr}(\tau )\text{I},$$

where τ=Jσ is the Kirchhoff stress (σ is the Cauchy stress and J is current volume/reference volume), $\epsilon^{e}$ is the elastic strain (based on the logarithmic strain), E is Young’s modulus and ν is Poisson’s ratio. Also, tr( ) denotes the trace and I denotes the identity tensor.

The creep part of the rate of deformation tensor, $\text{D}=\mathrm{sym}(\dot{F}\cdot F^{-1})$, is

2.5 $${\text{D}}^{c}=\frac{3}{2}{\dot{\epsilon }}_{c}\frac{\tau^{\prime }}{\sigma_{e}}=\frac{3}{2}\alpha \sigma_{e}^{n-1}\tau^{\prime },$$

with

2.6 $${\dot{\epsilon }}_{c}={\dot{\epsilon }}_{0}{\left(\frac{\sigma_{e}}{\sigma_{0}}\right)}^{n}=\alpha {\sigma_{e}}^{n}$$

and

2.7 $$\tau^{\prime }=\tau -\frac{1}{3}\mathrm{tr}(\tau )\text{I}\;\mathrm{and}\;\sigma_{e}=\sqrt{\frac{3}{2}\tau^{\prime }:\tau^{\prime }},$$

where n is the creep exponent, ${\dot{\epsilon }}_{0}$ is a reference strain rate, $\sigma_{0}$ is a reference stress and $\alpha ={\dot{\epsilon }}_{0}/\sigma_{0}^{n}$ is the power law creep pre-exponential factor. Also, the effective creep strain $\epsilon_{c}$ is given by $\epsilon_{c}=\int_{0}^{t}{\dot{\epsilon }}_{c}dt$ and t is time.

## 3. Bayesian-type statistical approach

The equations of the Bayesian-type statistical approach used to infer the creep parameters n, $\sigma_{0}$ and ${\dot{\epsilon }}_{0}$ from an indentation depth versus time response, from a residual surface profile or from a combination of these are presented here. A more complete presentation, background on the methodology and references are given in [15].

The ‘experimental’ indentation data consist of: (i) a vector characterizing the residual surface profile, $s^{m}$; and (ii) a vector characterizing the indentation depth versus time response, $h^{m}$. The components of the vector $s_{k}^{m}$, $k=1,\ldots ,K_{s}$ are values of the normalized surface coordinate, $z_{k}/h_{\mathrm{ref}}$ ($h_{\mathrm{ref}}$ is a conveniently chosen reference length) at specified values of normalized radial coordinate $r_{k}/h_{\mathrm{ref}}$. The components of the vector $h_{k}^{m}$, $k=1,\ldots ,K_{h}$ are values of the normalized indentation depth $h_{k}/h_{\mathrm{ref}}$ at specified values of normalized time $t_{k}/t_{\mathrm{ref}}$.

Finite-element solutions for a normalized residual surface profile, denoted by $s^{i}$, and for a normalized indentation depth versus time response, denoted by $h^{i}$, are used to construct a coarse database of indentation responses, with $i=1,2,\ldots ,K_{\mathrm{total}}$ and $K_{\mathrm{total}}$ is the total number of indentation response pairs ($s^{i}$,$h^{i}$) in the database. In practice, it is expected that there will be a delay between unloading and measuring the surface profile. The measured surface profile will, at least to some extent, depend on this delay which, if specified, can be incorporated into the formulation. However, for simplicity and because a standard delay time remains to be established, the database here is constructed using the surface profile immediately after unloading.

Treating the indentation depth versus time data and the surface profile data as being independent, the posterior probability $p(s^{i},h^{i}|s^{m},h^{m})$ associated with the ‘experimental’ data $\left(s^{m},h^{m}\right)$ is given by

3.1 $$p(s^{i},h^{i}|s^{m},h^{m})=\frac{p(s^{i}|s^{m})p(h^{i}|h^{m})}{Z_{\mathrm{sh}}},$$

where

3.2 $$p(s^{i}|s^{m})=\frac{p(s^{m}|s^{i})p(s^{i})}{Z_{s}}\;\mathrm{and}\;p(h^{i}|h^{m})=\frac{p(h^{m}|h^{i})p(h^{i})}{Z_{h}}.$$

In equations (3.1) to (3.2), there is no sum on i.

The constants $Z_{s}$, $Z_{h}$ and $Z_{\mathrm{sh}}$, which ensure that the posterior probability values lie in the range 0 to 1, are given by

3.3 $$Z_{s}=\sum_{i=1}^{K_{\mathrm{total}}}p(s^{m}|s^{i})p(s^{i}),\;Z_{h}=\sum_{i=1}^{K_{\mathrm{total}}}p(h^{m}|h^{i})p(h^{i}),\;Z_{\mathrm{sh}}=\sum_{i=1}^{K_{\mathrm{total}}}p(s^{i}|s^{m})p(h^{i}|h^{m}).$$

The likelihood functions, which measure the difference between the ‘experimental’ data and the predicted responses in the database, are (see Zhang *et al.* [15])

3.4 $$p(s^{m}|s^{i})=(\frac{1}{{\hat{\xi }}_{s}^{i}\sqrt{2\pi }}{)}^{K_{s}}\exp(-\frac{K_{s}}{2})\;\mathrm{and}\;p(h^{m}|h^{i})=(\frac{1}{{\hat{\xi }}_{h}^{i}\sqrt{2\pi }}{)}^{K_{h}}\exp(-\frac{K_{h}}{2}),$$

where $K_{s}$ is the number of data points on the residual surface profile curve, $K_{h}$ is the number of data points on the indentation depth versus time curve and the variances ${\left({\hat{\xi }}_{s}^{i}\right)}^{2}$ and ${\left({\hat{\xi }}_{h}^{i}\right)}^{2}$ are given by the maximum likelihood estimates

3.5 $${\left({\hat{\xi }}_{s}^{i}\right)}^{2}=\frac{1}{K_{s}}\sum_{k=1}^{K_{s}}{\left(s_{k}^{m}-s_{k}^{i}\right)}^{2}\;\mathrm{and}\;{\left({\hat{\xi }}_{h}^{i}\right)}^{2}=\frac{1}{K_{h}}\sum_{k=1}^{K_{h}}{\left(h_{k}^{m}-h_{k}^{i}\right)}^{2},$$

where the subscript k denotes the kth component of each vector. If one of the variances in equation (3.5) is equal to 0, its corresponding likelihood in equation (3.4) is infinite and the corresponding posterior probability is set to 1.

For all sets of creep parameters in the database, a uniform prior is used for both $p(s^{i})$ and $p(h^{i})$ in equation (3.2). Outside the range of values in the database, $p(s^{i})=0$ and $p(h^{i})=0$. The posterior probabilities are evaluated by substituting the prior values and the likelihood values from equation (3.4) into equation (3.2).

## 4. Material parameters

The ‘experimental’ materials considered are : (i) amorphous selenium (Se) at $35^{\circ }$C; (ii) solid acid ${\mathrm{CsHSO}}_{4}$ at $145^{\circ }$C; and (iii) tin (Sn) at $129^{\circ }$C. The values of the material parameters characterizing these materials are given in table 1.

**Table 1.** Constitutive parameters characterizing the three input ‘experimental’ materials.

	E(GPa)	ν	n	$\sigma_{0}$(MPa)	${\dot{\epsilon }}_{0}(s^{-1})$	$\alpha ({\mathrm{Pa}}^{-n}s^{-1})$
Se ($35^{\circ }C$)	9.2	0.33	1.15	8.740	$1.0\times 10^{-4}$	$1.04\times 10^{-12}$
${\mathrm{CsHSO}}_{4}$ ($145^{\circ }C$)	1.2	0.33	3.59	0.01512	1.0	$9.89\times 10^{-16}$
Sn ($129^{\circ }C$)	45	0.33	6.60	9.330	1.0	$9.97\times 10^{-47}$

For Se, the values of E, n and α are taken from [10], and the value of Poisson’s ratio ν is from [20]. For ${\mathrm{CsHSO}}_{4}$, the values of n and α are taken from table 1 of [21]. The value of E is obtained by a linear fit to the uniaxial data at a strain rate of $10^{-2}\;s^{-1}$ up to a stress of 6.0 MPa in fig. 1(a) of [21]. For Sn, the value of n is taken from [22] and the value of α is obtained by a fit to data in fig. 2(b) of [22]. The value of E is taken to be 45 GPa [23] and the value of ν is taken from [24].

## 5. Indentation responses

### (a) Constant load and hold indentation creep

The imposed loading history models a constant load and hold indentation creep test, with the magnitude of the applied force on the indenter, f(t) in equation (2.1), prescribed to be

5.1 $$f(t)=\left\{ \begin{array}{ll} \zeta h_{\mathrm{ref}}^{2}\sigma_{0}t/t_{1}, & 0\leq t\leq t_{1}\\ \zeta h_{\mathrm{ref}}^{2}\sigma_{0}, & t_{1}<t\leq t_{2}\\ \zeta h_{\mathrm{ref}}^{2}\sigma_{0}(t_{3}-t)/(t_{3}-t_{2}), & t_{2}<t\leq t_{3} \end{array}\right.$$

where the rise time is $t_{1}{\dot{\epsilon }}_{0}=10^{-4}$, the hold time is $t_{2}{\dot{\epsilon }}_{0}=1.0$, the load release time, $t_{3}-t_{2}$, is given by $t_{3}{\dot{\epsilon }}_{0}=t_{2}{\dot{\epsilon }}_{0}+10^{-4}$ and the normalizing length is taken to be $h_{\mathrm{ref}}=3.43\times 10^{-4}L_{0}$ in all calculations. The value of $\sigma_{0}$ used in equation (5.1) for each material is given in table 1. The value of the non-dimensional factor ζ is selected, so that the indentation depth h is large compared with the finite-element size near the indenter but with the large strain gradients confined to the region with the finest finite-element resolution. The values of ζ used in the calculations are given in appendix A.

For power-law creep with elastic strains neglected, equation (2.6), Bower *et al.* [1] derived a relation for normalized indentation depth rate:

5.2 $${\dot{\epsilon }}_{\mathrm{crp}}=\frac{1}{h_{\mathrm{crp}}}\frac{dh_{\mathrm{crp}}}{dt}=\beta p^{n}$$

with

5.3 $$p=\frac{f(t)}{\pi h_{\mathrm{crp}}^{2}\tan^{2}\gamma },$$

where ${\dot{\epsilon }}_{\mathrm{crp}}$ is indentation strain rate, p is the nominal contact pressure (contact force/contact area) (see figure 1), and β is an indentation creep parameter.

For $f(t)\equiv f_{\mathrm{const}}$, integration of equation (5.2) with respect to t gives

5.4 $$h_{\mathrm{crp}}={\left(2n\beta \right)}^{1/2n}(\frac{f_{\mathrm{const}}}{\pi \tan^{2}\gamma }{)}^{1/2}t^{1/2n}.$$

Note that since the force magnitude is prescribed constant, both the indentation pressure, p, and the indentation strain rate, ${\dot{\epsilon }}_{\mathrm{crp}}$, vary with time.

For an elastic solid, the relation between indentation depth h and indentation force $f_{\mathrm{const}}$ in the axisymmetric Boussinesq problem is given by Sneddon [25]:

5.5 $$h_{\mathrm{ela}}={\left(\frac{\pi f_{\mathrm{const}}}{2E^{*}\tan\gamma }\right)}^{1/2},$$

with $E^{*}=E/(1-\nu^{2})$.

As exploited by Su *et al.* [10], the indentation depths induced by a constant load for a power-law creeping solid, equation (5.4), and for an elastic solid, equation (5.5), are each proportional to $\sqrt{f_{\mathrm{const}}}$ so that in the power-law creep regime

5.6 $$\frac{h_{\mathrm{crp}}}{h_{\mathrm{ela}}}={\left(2n\beta \right)}^{1/2n}(\frac{2E^{*}}{\pi^{2}\tan\gamma }{)}^{1/2}t^{1/2n}.$$

Hence, the ratio $h/h_{\mathrm{ela}}$ is independent of $f_{\mathrm{const}}$ both at the beginning of indentation when $h_{\mathrm{ela}}$ dominates and at steady-state creep when $h_{\mathrm{crp}}$ dominates. Thus, $h_{\mathrm{ela}}$ provides a natural choice of reference length [10]. Attention here is confined to scaling relations associated with load and hold indentation, but we note that scaling relations for other loading histories have been given in [5,10].

The values of $h_{\mathrm{ela}}$ are $h_{\mathrm{ela},\mathrm{Se}}=7.88\times 10^{-4}L_{0}$ for Se, $h_{\mathrm{ela},{\mathrm{CsHSO}}_{4}}=3.43\times 10^{-4}L_{0}$ for ${\mathrm{CsHSO}}_{4}$ and $h_{\mathrm{ela},\mathrm{Sn}}=1.18\times 10^{-3}L_{0}$ for Sn. If we take $f_{\mathrm{const}}=100\;\mathrm{mN}$, then $h_{\mathrm{ela}}$ is 2.33 μm, 6.46 μm and 1.06 μm for Se, ${\mathrm{CsHSO}}_{4}$ and Sn, respectively. For each of the three materials its value of $h_{\mathrm{ela}}$ is used as the reference length.

In their experiments Su *et al.* [10] found that the $h/h_{\mathrm{ela}}$ versus t response for amorphous selenium at $35^{\circ }C$ under various applied indentation forces collapsed onto a single curve even in the transient regime. Here, calculations with indentation forces of 1/3 and 2 times the f(t) value in equation (5.1) were carried out for Se (n=1.15), ${\mathrm{CsHSO}}_{4}$ (n=3.59) and Sn (n=6.60), and the calculated curves of $h/h_{\mathrm{ela}}$ versus t collapsed onto a single curve.

### (b) Finite-element implementation

The reference finite-element mesh for the configuration in figure 1 consists of 8100 nodes, corresponding to 7921 four-node bilinear axisymmetric quadrilateral elements. In a $0.1L_{0}\times 0.1L_{0}$ fine mesh region near the indenter tip, 60×60 elements are used with a uniform square element size $(1.7\times 10^{-3})L_{0}\times (1.7\times 10^{-3})L_{0}$. Thus, the element size in the fine mesh region is $2.2h_{\mathrm{ela},\mathrm{Se}}$ for Se, $5.0\;h_{\mathrm{ela},{\mathrm{CsHSO}}_{4}}$ for ${\mathrm{CsHSO}}_{4}$ and $1.4\;h_{\mathrm{ela},\mathrm{Sn}}$ for Sn. The element size is gradually increased outside the uniform meshed region. Reduced integration with hourglass control is used. Also, the error tolerance in ABAQUS [19] standard is set to $10^{-3}$. More details on the ABAQUS [19] indentation implementation used are given in [26].

Convergence was investigated using a refined mesh with 1/4 the element sizes of the reference mesh, giving 31 684 quadrilateral elements and 32 041 nodes. For all three materials, the indentation depth versus time responses calculated with the two meshes essentially coincided. The differences between indentation depths when $t{\dot{\epsilon }}_{0}>10^{-4}$ were less than 2.7%, 0.2% and 0.1% for Se, ${\mathrm{CsHSO}}_{4}$ and Sn, respectively. The residual surface profile for Se involved sink-in with a maximum profile difference of 0.4%, while the surface profiles for ${\mathrm{CsHSO}}_{4}$ and Sn involved pile-up with a maximum pile-up height difference of 1.8% between the two meshes. Also, the maximum indentation depths at non-dimensional time $t{\dot{\epsilon }}_{0}=1.0$ differed by less than 0.1%. All results to be presented subsequently were obtained using the reference finite-element mesh.

### (c) ‘Experimental’ indentation responses

**Figure 2.**
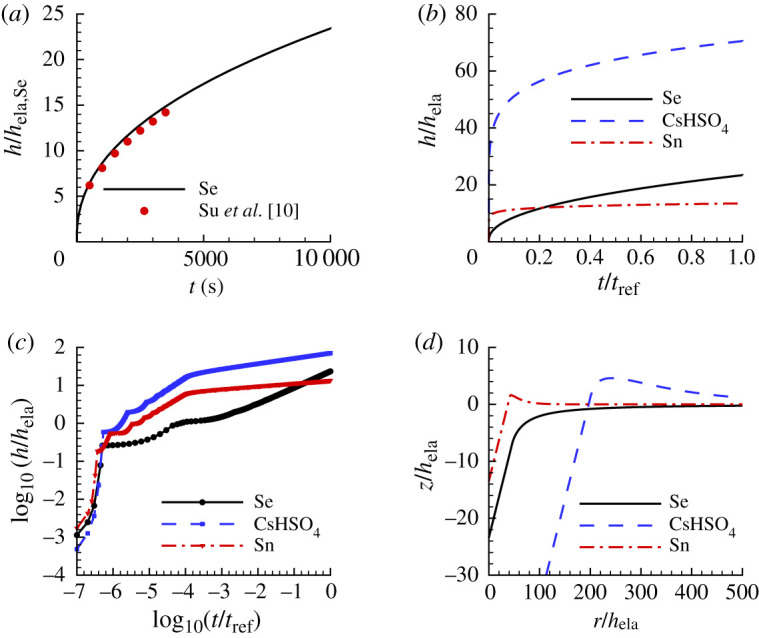
The indentation responses of the three ‘experimental’ materials, Se, ${\mathrm{CsHSO}}_{4}$ and Sn, in constant load and hold indentation; see equation (5.1). The material parameters are given in table 1. (*a*) Normalized indentation depth $h/h_{\mathrm{ela},\mathrm{Se}}$ versus time t for Se. The points are data taken from Su *et al.* [10]. (*b*) $h/h_{\mathrm{ela}}$ versus $t/t_{\mathrm{ref}}$. (*c*) $\log_{10}(h/h_{\mathrm{ela}})$ versus $\log_{10}(t/t_{\mathrm{ref}})$. (*d*) Normalized surface profiles near the indenter after unloading. The values of $h_{\mathrm{ela}}$ and $t_{\mathrm{ref}}=1/{\dot{\epsilon }}_{0}$ in (*b*–*d*) are specific to each material. (Online version in colour.)

Figure 2*a* shows the computed normalized indentation depth, $h/h_{\mathrm{ela},\mathrm{Se}}$, versus time, t, response obtained using material parameters for Se in table 1. The points are experimentally measured data from fig. 6(b) of Su *et al.* [10] and show that the experimental and computed responses are in very good agreement.

Figure 2*b* shows $h/h_{\mathrm{ela}}$ versus $t/t_{\mathrm{ref}}$ responses for three sets of material parameters in table 1, Se, ${\mathrm{CsHSO}}_{4}$ and Sn, when $t_{\mathrm{ref}}=1/{\dot{\epsilon }}_{0}$ and $h_{\mathrm{ela}}$ is taken to be the specified value for each of the three materials. Figure 2*c* shows a $\log_{10}-\log_{10}$ plot of the data in figure 2*b*. Note that the value of $h/h_{\mathrm{ela}}$ at which each material enters steady-state creep differs. The unloading parts of the responses are not shown in figure 2*a*–*c*, and are not used for identifying the power-law creep parameters.

In the early stages of indentation, the plot of indentation depth h versus time t is not smooth because when a new node comes into contact with the indenter, the contact length increases by the length of one-element. This discrete change in contact length occurs in the early stages of indentation when both t and h are small. In contrast to [15], the finite-element output responses are not smoothed, since only the differences between the ‘experimental’ input response and the responses of sets of material property values in the database matter, as described in §3.

Figure 2*d* shows the normalized surface profiles near the indenter after unloading for the three materials. The residual surface profile of ${\mathrm{CsHSO}}_{4}$ (dashed line) has a larger normalized indentation depth than those for Se (solid line) and Sn (dash dot line). The residual surface profile for Se exhibits sink-in while those for ${\mathrm{CsHSO}}_{4}$ and Sn exhibit pile-up.

**Figure 3.**
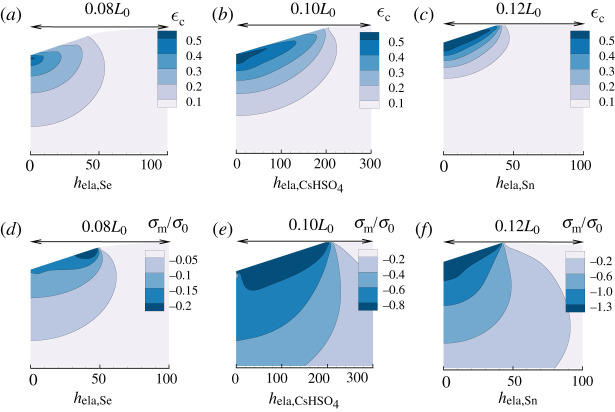
Distributions of effective creep strain, $\epsilon_{c}$, and mean normal stress, $\sigma_{m}$, in the vicinity of the indenter at $t{\dot{\epsilon }}_{0}=1.0$ (where ${\dot{\epsilon }}_{0}$ is the value in table 1 for each material). The indentation depths are 23.4$h_{\mathrm{ela},\mathrm{Se}}$, $70.5h_{\mathrm{ela},{\mathrm{CsHSO}}_{4}}$ and 13.5$h_{\mathrm{ela},\mathrm{Sn}}$ for Se, ${\mathrm{CsHSO}}_{4}$ and Sn, respectively. (*a*) Distribution of $\epsilon_{c}$ for Se. (*b*) Distribution of $\epsilon_{c}$ for ${\mathrm{CsHSO}}_{4}$. (*c*) Distribution of $\epsilon_{c}$ for Sn. (*d*) Distribution of $\sigma_{m}/\sigma_{0}$ for Se. (*e*) Distribution of $\sigma_{m}/\sigma_{0}$ for ${\mathrm{CsHSO}}_{4}$. (*f* ) Distribution of $\sigma_{m}/\sigma_{0}$ for Sn.

Figure 3 shows distributions of effective creep strain, $\epsilon_{c}$, and mean normal stress, $\sigma_{m}$, for the ‘experimental’ materials subject to constant load and hold loading in the vicinity of the indenter at $t_{2}{\dot{\epsilon }}_{0}=1.0$ in equation (5.1). The size scale of the regions shown is material dependent, being $100h_{\mathrm{ela},\mathrm{Se}}$, $300h_{\mathrm{ela},{\mathrm{CsHSO}}_{4}}$ and $100h_{\mathrm{ela},\mathrm{Sn}}$ for Se, ${\mathrm{CsHSO}}_{4}$ and Sn in figure 3*a*–*c*, respectively. For each of the three materials, the state of deformation shown is at the maximum indentation depth $h_{\max}$ for each material just before unloading is initiated. For Se $h_{\max}=0.0185L_{0}=23.4h_{\mathrm{ela},\mathrm{Se}}$, for ${\mathrm{CsHSO}}_{4}\;h_{\max}=0.0241L_{0}=70.5h_{\mathrm{ela},{\mathrm{CsHSO}}_{4}}$ and for Sn $h_{\max}=0.0159L_{0}=13.5h_{\mathrm{ela},\mathrm{Sn}}$.

The extent, in terms of $h_{\mathrm{ela},{\mathrm{CsHSO}}_{4}}$ of the region with relatively large values of $\epsilon_{c}$ for ${\mathrm{CsHSO}}_{4}$ is much larger, $\approx 300h_{\mathrm{ela},{\mathrm{CsHSO}}_{4}}$, than is the extent of the corresponding regions in terms of $h_{\mathrm{ela}}$ for Se and Sn. This is because the ratio $\sigma_{0}/E$ for ${\mathrm{CsHSO}}_{4}$ is more than one order of magnitude smaller than for the other two materials (table 1). The creep deformations for Sn are more localized under the indenter than for Se and ${\mathrm{CsHSO}}_{4}$ because Sn has a larger value of n and a smaller value of α.

Figure 3*d*–*f* shows the contours of the corresponding mean normal stress $\sigma_{m}/\sigma_{0}$ for Se, ${\mathrm{CsHSO}}_{4}$ and Sn. The mean normal stress is given by

5.7 $$\sigma_{m}=\frac{1}{3}\mathrm{tr}(\sigma ),$$

where σ is the Cauchy stress tensor (since the materials are nearly incompressible there is little difference between the mean normal stress values based on Cauchy stress and based on Kirchhoff stress). The peak magnitude of $\sigma_{m}/\sigma_{0}$ is much smaller for Se than for ${\mathrm{CsHSO}}_{4}$ and Sn. For Se, the value of n is the smallest for the three materials and the value of α is the largest.

### (d) Construction of the databases

The creep exponent n and associated pre-exponential factor α in equation (2.6) define the power-law creep response. However, since the dimensions of α are ${\mathrm{stress}}^{-n}/\mathrm{time}$, it is not convenient to base the databases needed for the Bayesian analysis on α. Hence, for each material, the databases are constructed for the parameters n, $\sigma_{0}$ and ${\dot{\epsilon }}_{0}$.

For each of the three ‘experimental’ materials in table 1, one database was constructed consisting of indentation depth versus time responses and residual surface profiles directly calculated from finite-element simulation. All the database indentation responses, $h^{i}$ with $K_{h}=64$ data points and $s^{i}$ with $K_{s}=56$ data points, where $i=1,\ldots ,K_{\mathrm{total}}$, are evaluated at specified values of ${\dot{\epsilon }}_{0}t$ and $r/h_{\mathrm{ela}}$ that are obtained by interpolation of the computed responses. The specified values of ${\dot{\epsilon }}_{0}t$ and $r/h_{\mathrm{ela}}$ are distributed in a material dependent nonuniform manner because of the large variation in time scales and length scales between the three materials. For the calculation of the likelihood functions, equations (3.4), and of the variances, equation (3.5), the ‘experimental’ indentation responses, $h^{m}$ and $s^{m}$, are evaluated at the same points.

In all three databases, the creep exponent n∈[1.0,7.0] with step size 0.1 (61 points) and ${\dot{\epsilon }}_{0}t_{2}\in [0.1,1.0,10.0,100.0]$ (4 points). For Se and Sn, $\sigma_{0}/E\in [1\times 10^{-4},1.1\times 10^{-3}]$ with step size $1\times 10^{-4}$ (11 points) while for ${\mathrm{CsHSO}}_{4}$, $\sigma_{0}/E\in [1\times 10^{-5},1.1\times 10^{-4}]$ with step size $1\times 10^{-5}$ (11 points). Thus there are $K_{\mathrm{total}}=2684$ sets of parameter values in each of the three databases. For each set of parameter values, one finite-element calculation was carried out.

As in [15,26,27], databases obtained directly from the finite-element calculations are relatively coarse and interpolation is used to populate finer databases. Here, linear interpolation between nearby material parameters associated with database ‘points’ (each database ‘point’ consists of a vector of indentation depth versus time and a vector of surface profile points) in the coarse databases was used to define the responses associated with the ‘points’ in the finer databases. The interpolated finer databases have a step size of 0.02 in n and of 0.2 in $\log_{10}({\dot{\epsilon }}_{0}t_{2})$ for all three materials, of $0.2\times 10^{-4}$ in $\sigma_{0}/E$ for Se and Sn, and of $0.2\times 10^{-5}$ in $\sigma_{0}/E$ for ${\mathrm{CsHSO}}_{4}$. This results in $K_{\mathrm{total}}=245\;616$ points in the finer databases. The interpolated databases are used for the predictions of creep parameters.

The accuracy of the interpolation was checked by carrying out a few finite-element calculations using interpolated values of material parameters. The agreement between calculated and interpolated responses was best for larger values of the creep stress exponent n and worse for values of n near 1. However, as the results to be presented subsequently will show, the lack of accuracy of the interpolated response for n≈1 does not adversely affect the ability to predict the indentation creep response and the associated power-law creep parameters.

## 6. Identification of power-law creep properties from indentation

Values of the creep material parameters n, $\sigma_{0}$ and ${\dot{\epsilon }}_{0}$ are obtained from the indentation responses. The predicted material parameters are then used to calculate the spatially uniform uniaxial creep and relaxation responses from a one-element finite-element solution.

For uniaxial creep loading the prescribed stress σ is

6.1 $$\sigma =\left\{ \begin{array}{ll} \frac{\sigma_{a}t}{t_{C1}}, & 0\leq t\leq t_{C1}\\ \sigma_{a}, & t_{C1}<t\leq t_{C2}, \end{array}\right.$$

where $\sigma_{a}=0.5\sigma_{0}=4.37\times 10^{6}\;\mathrm{Pa}$, $t_{C1}=10^{-4}\;s$, $t_{C2}=3000\;s$ for Se, $\sigma_{a}=0.1\sigma_{0}=0.1512\times 10^{4}\;\mathrm{Pa}$, $t_{C1}=10^{-7}\;s$, $t_{C2}=500\;s$ for ${\mathrm{CsHSO}}_{4}$ and $\sigma_{a}=0.2\sigma_{0}=1.866\times 10^{6}\;\mathrm{Pa}$, $t_{C1}=10^{-5}\;s$, $t_{C2}=4000\;s$ for Sn, giving the strain rate values $\alpha \sigma_{a}^{n}$ to be $4.51\times 10^{-5}\;s^{-1}$, $2.57\times 10^{-4}\;s^{-1}$ and $2.44\times 10^{-5}\;s^{-1}$ for Se, ${\mathrm{CsHSO}}_{4}$ and Sn, respectively. The value of $\sigma_{0}$ is for each material given in table 1.

For an imposed $\sigma_{a}$ at t=0 (i.e. with the rise time neglected),

6.2 $$\epsilon =\frac{\sigma_{a}}{E}+\alpha \sigma_{a}^{n}t.$$

For uniaxial stress relaxation loading, the displacement rate is prescribed so that $\epsilon =\ln(\ell /\ell_{0})$ is a constant, where ℓ is the current length and $\ell_{0}$ the initial length, and is given by

6.3 $$\epsilon =\left\{ \begin{array}{ll} \frac{\epsilon_{a}t}{t_{R1}}, & 0\leq t\leq t_{R1}\\ \epsilon_{a}, & t_{R1}<t\leq t_{R2}, \end{array}\right.$$

where $\epsilon_{a}=1\times 10^{-7}$, $t_{R1}=10^{-3}\;s$, $t_{R2}=150\;s$ for Se, $\epsilon_{a}=5\times 10^{-8}$, $t_{R1}=10^{-14}\;s$, $t_{R2}=500\;s$ for ${\mathrm{CsHSO}}_{4}$ and $\epsilon_{a}=1\times 10^{-5}$, $t_{R1}=10^{-11}\;s$, $t_{R2}=2\times 10^{4}\;s$ for Sn.

For an imposed $\epsilon_{a}$ at t=0, and with n>1,

6.4 $$\sigma =\frac{E\epsilon_{a}}{{\left[1+\alpha {\left(E\epsilon_{a}\right)}^{n}(n-1)\epsilon_{a}^{-1}t\right]}^{1/(n-1)}}=\frac{\sigma_{a}}{{\left[1+\alpha \sigma_{a}^{n}(n-1)\epsilon_{a}^{-1}t\right]}^{1/(n-1)}}.$$

Note that with n>1, $\sigma_{a}^{n}/\epsilon_{a}={\left(E\epsilon_{a}\right)}^{n}/\epsilon_{a}=0$ for $\epsilon_{a}=0$. Also, in both equations (6.2) and (6.4), the response is governed by $\alpha \times {\left(\mathrm{stress}\;\mathrm{quantity}\right)}^{n}$.

A significant difference between the indentation depth versus time response in equation (5.6) and the uniaxial creep responses in equations (6.2) and (6.4) is that $h_{\mathrm{crp}}/h_{\mathrm{ela}}$ is independent of the load magnitude (i.e. $h_{\mathrm{crp}}$ and $h_{\mathrm{ela}}$ have the same dependence on applied load) whereas the uniaxial creep responses strongly depend on the applied load magnitude.

### (a) Bayesian identification

For the three ‘experimental’ materials in table 1, the set of values n, $\sigma_{0}$ and ${\dot{\epsilon }}_{0}$ with the largest posterior probability is identified as the set of parameter values characterizing the creep response of the ‘experimental’ material. The value of the pre-exponential factor α is then calculated using equation (2.6).

Once the initial database is constructed, the computations for the interpolation and for the statistical analysis are very light and are quickly carried out on a personal computer [26].

#### (i) Noise-free data

For each database, the posterior probability distribution is calculated from: (i) indentation depth versus time data (HT); (ii) residual surface profile data (S); and (iii) both indentation depth versus time data and residual surface profile data (HTS). The values of n, $\sigma_{0}$ and ${\dot{\epsilon }}_{0}$ associated with the largest posterior probability value obtained from (i), (ii), (iii) and the responses based on these values are denoted by HT, S, HTS, respectively.

**Figure 4.**
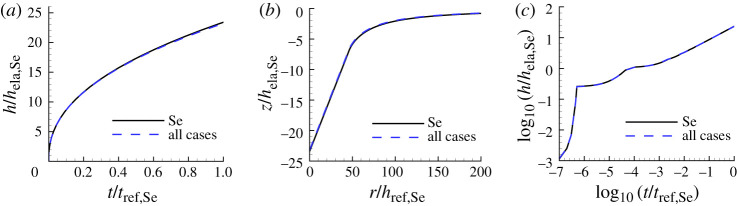
Comparison of predicted and ‘experimental’ indentation responses for Se. The indentation responses with the largest value of posterior probability for the indentation depth versus time data (HT), for the residual surface profile data (S) and for both the indentation depth versus time data and the residual surface profile data (HTS) coincide and are denoted by ‘all cases’. The associated values of n, $\sigma_{0}$, ${\dot{\epsilon }}_{0}$ and α are given in table 2. (*a*) Normalized indentation depth $h/h_{\mathrm{ela},\mathrm{Se}}$ versus normalized time $t/t_{\mathrm{ref},\mathrm{Se}}$ where $t_{\mathrm{ref},\mathrm{Se}}=1/{\dot{\epsilon }}_{0,\mathrm{Se}}$. (*b*) Normalized surface profiles, $z/h_{\mathrm{ela},\mathrm{Se}}$ versus $r/h_{\mathrm{ela},\mathrm{Se}}$, near the indenter after unloading. (*c*) $\log_{10}$–$\log_{10}$ plot of (*a*). On the scales in this figure, the ‘all cases’ predictions are indistinguishable from the corresponding ‘experimental’ responses.

For Se, the predicted values of n, $\sigma_{0}$, ${\dot{\epsilon }}_{0}$ and therefore α using any of the three sets of data (HT, S and HTS) coincide. Figure 4 shows the indentation responses (dashed lines and labelled ‘all cases’) obtained using these predicted parameter values. For comparison, the indentation responses using the input properties of Se in table 1 (solid lines) are also shown. The indentation responses of ‘all cases’ are nearly indistinguishable from the ‘experimental’ indentation responses.

**Table 2.** Predicted values of n, $\sigma_{0}$, ${\dot{\epsilon }}_{0}$, α and the associated largest value of posterior probability $p_{1}$ for Se obtained based on noise-free ‘experimental’ indentation responses. The predicted values obtained using the indentation depth versus time data (HT), using the residual surface profile data (S) and using both the indentation depth versus time data and the residual surface profile data (HTS) all coincide and are denoted by ‘all cases’.

	n	$\sigma_{0}$(MPa)	${\dot{\epsilon }}_{0}(s^{-1})$	$\alpha \;({\mathrm{Pa}}^{-n}\;s^{-1})$	$p_{1}$
all cases	1.16	8.648	$1.0\times 10^{-4}$	$0.898\times 10^{-12}$	1.00

The predicted parameter values n, $\sigma_{0}$, ${\dot{\epsilon }}_{0}$, α and associated largest posterior probability values $p_{1}$ using three types of data based on the noise-free ‘experimental’ indentation responses of Se in figure 2 are given in table 2.

The predicted parameter values of n and α are the same for all three cases and are close to the input values but a direct comparison of the values of α is not meaningful unless the values of n coincide since the units of α vary with n.

**Figure 5.**
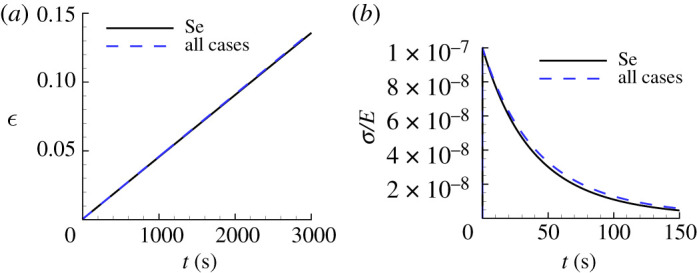
Comparison of the predicted uniaxial creep and stress relaxation responses using the ‘all cases’ parameter values in table 2 with the corresponding ‘experimental’ responses for Se. (*a*) Uniaxial logarithmic strain, ϵ, versus time, t. (*b*) Normalized uniaxial Cauchy stress, σ/E, versus time, t. On the scales in (*a*), the ‘all cases’ prediction is indistinguishable from the corresponding ‘experimental’ response.

The uniaxial creep responses obtained from a one-element finite-element uniaxial solution with the loading given by equation (6.1) for the ‘all cases’ parameter values in table 2 are shown in figure 5*a*. The corresponding stress relaxation responses using equation (6.3) are shown in figure 5*b*. In both figures, the predicted responses compare well with those obtained using the input material parameter values for Se in table 1.

**Figure 6.**
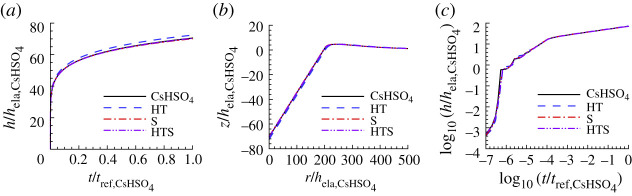
Comparison of predicted and ‘experimental’ indentation responses for ${\mathrm{CsHSO}}_{4}$. The indentation responses are those corresponding to the largest value of posterior probability for the indentation depth versus time data (HT), for the residual surface profile data (S) and for both the indentation depth versus time data and the residual surface profile data (HTS). The associated values of n, $\sigma_{0}$, ${\dot{\epsilon }}_{0}$ and α are given in table 3. (*a*) Normalized indentation depth $h/h_{\mathrm{ela},{\mathrm{CsHSO}}_{4}}$ versus normalized time $t/t_{\mathrm{ref},{\mathrm{CsHSO}}_{4}}$ where $t_{\mathrm{ref},{\mathrm{CsHSO}}_{4}}=1/{\dot{\epsilon }}_{0,{\mathrm{CsHSO}}_{4}}$. (*b*) Normalized surface profiles, $z/h_{\mathrm{ela},{\mathrm{CsHSO}}_{4}}$ versus $r/h_{\mathrm{ela},{\mathrm{CsHSO}}_{4}}$, near the indenter after unloading. (*c*) $\log_{10}$–$\log_{10}$ plot of (*a*). On the scales in this figure, the predictions with S and HTS data are indistinguishable from the corresponding ‘experimental’ responses.

Figure 6 shows the indentation responses calculated using the creep properties for ${\mathrm{CsHSO}}_{4}$ with the largest value of posterior probability $p_{1}$ compared with the ‘experimental’ indentation responses. As seen in figure 6*a*, for ${\mathrm{CsHSO}}_{4}$, the representation of the indentation depth versus time response is improved by considering surface profile data. However, the improvement is small and is negligible for the $\log_{10}-\log_{10}$ plot in figure 6*c*.

**Table 3.** Predicted values of n, $\sigma_{0}$, ${\dot{\epsilon }}_{0}$, α and the associated largest value of posterior probability $p_{1}$ for ${\mathrm{CsHSO}}_{4}$ obtained based on noise-free ‘experimental’ indentation responses. See the caption of figure 6 for the meanings of HT, S and HTS.

	n	$\sigma_{0}$(MPa)	${\dot{\epsilon }}_{0}(s^{-1})$	$\alpha ({\mathrm{Pa}}^{-n}s^{-1})$	$p_{1}$
HT	3.58	0.0312	15.8	$12.9\times 10^{-16}$	1.00
S	3.66	0.0528	100	$5.19\times 10^{-16}$	0.59
HTS	3.58	0.0552	100	$10.6\times 10^{-16}$	1.00

**Figure 7.**
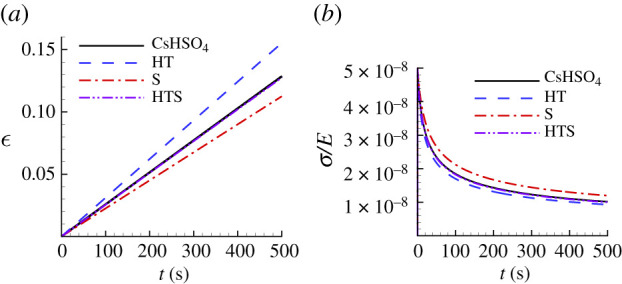
Comparison of the predicted uniaxial creep and stress relaxation responses using the parameter values in table 3 with the corresponding ‘experimental’ responses for ${\mathrm{CsHSO}}_{4}$. See the caption of figure 6 for the meanings of HT, S and HTS. (*a*) Uniaxial logarithmic strain, ϵ, versus time, t. (*b*) Normalized uniaxial Cauchy stress, σ/E, versus time, t. On the scales in this figure, the predictions with HTS data are indistinguishable from the corresponding ‘experimental’ responses.

Table 3 shows the predicted parameter values for ${\mathrm{CsHSO}}_{4}$ and the value of the associated largest posterior probability obtained from the Bayesian analysis and figure 7 shows the comparison between the uniaxial creep and uniaxial stress relaxation responses using the predicted creep parameter values in table 3, for ${\mathrm{CsHSO}}_{4}$ and the ‘experimental’ responses. For ${\mathrm{CsHSO}}_{4}$, neither the parameter values based on fitting the indentation depth versus time response (HT) nor the residual surface profile (S) gives a particularly good fit to the uniaxial creep and stress relaxation responses but when both sets of data are used (HTS) an excellent fit is obtained. We note that the predicted values of reference strain rate for the HTS fit is a factor of 100 times the input value of ${\dot{\epsilon }}_{0}=1\;s^{-1}$. Nevertheless, the predicted values of n and α are very close to the input ‘experimental’ values.

**Figure 8.**
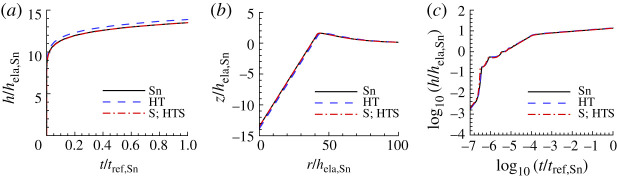
Comparison of predicted and ‘experimental’ indentation responses for Sn. See the caption of figure 6 for the meanings of HT, S and HTS. The associated values of n, $\sigma_{0}$, ${\dot{\epsilon }}_{0}$ and α are given in table 4. (*a*) Normalized indentation depth $h/h_{\mathrm{ela},\mathrm{Sn}}$ versus normalized time $t/t_{\mathrm{ref},\mathrm{Sn}}$ where $t_{\mathrm{ref},\mathrm{Sn}}=1/{\dot{\epsilon }}_{0,\mathrm{Sn}}$. (*b*) Normalized surface profiles, $z/h_{\mathrm{ela},\mathrm{Sn}}$ versus $r/h_{\mathrm{ela},\mathrm{Sn}}$, near the indenter after unloading. (*c*) $\log_{10}$–$\log_{10}$ plot of (*a*). On the scales in (*a*) and (*b*), the predictions with S; HTS data are indistinguishable from the corresponding ‘experimental’ responses. In (*c*), all three responses are indistinguishable.

Figure 8 shows the indentation responses calculated using the creep parameter values for Sn that have the largest value of posterior probability $p_{1}$. As for ${\mathrm{CsHSO}}_{4}$, the prediction of the indentation response of the ‘experimental’ material is slightly improved by considering surface profile data, figure 8*a*. For Sn, the creep parameter values in table 4 obtained using only surface profile data (S) and those obtained using both indentation depth versus time data and surface profile data (HTS) are identical. The HTS (or S) predicted value of ${\dot{\epsilon }}_{0}$ is a factor of 10 times the ‘experimental’ input value of ${\dot{\epsilon }}_{0}$ for Sn in table 1.

**Table 4.** Predicted values of n, $\sigma_{0}$, ${\dot{\epsilon }}_{0}$, α and the associated largest value of posterior probability $p_{1}$ for Sn obtained based on noise-free ‘experimental’ indentation responses. See the caption of figure 6 for the meanings of HT, S and HTS.

	n	$\sigma_{0}$(MPa)	${\dot{\epsilon }}_{0}(s^{-1})$	$\alpha ({\mathrm{Pa}}^{-n}s^{-1})$	$p_{1}$
${\mathrm{HT}}_{\;}$	6.64	10.80	3.98	$7.91\times 10^{-47}$	0.85
S	6.46	13.50	10	$86.7\times 10^{-47}$	1.00
HTS	6.46	13.50	10	$86.7\times 10^{-47}$	1.00

**Figure 9.**
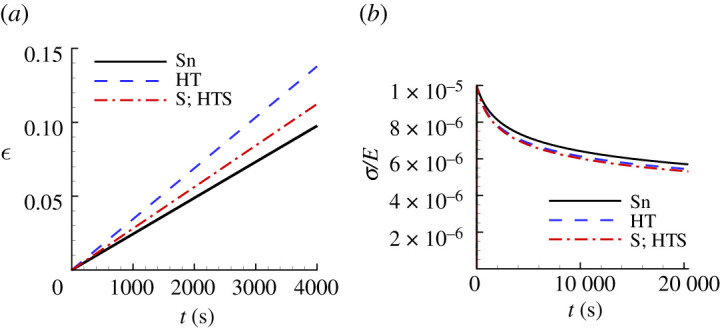
Comparison of the predicted uniaxial creep and stress relaxation responses using the parameter values in table 4 with the corresponding ‘experimental’ responses for Sn. See the caption of figure 6 for the meanings of HT, S and HTS. (*a*) Uniaxial logarithmic strain, ϵ, versus time, t. (*b*) Normalized uniaxial Cauchy stress, σ/E, versus time, t.

Figure 9 shows the uniaxial creep responses and uniaxial stress relaxation responses predicted for Sn using the creep parameter values in table 4 compared with the corresponding ‘experimental’ responses. Neither of the predicted responses for Sn in figure 9*a* provides a particularly good representation of the ‘experimental’ uniaxial creep response, although the inclusion of surface profile data does improve the prediction. On the other hand, both the HT and S; HTS relaxation responses in figure 9*b* do provide a fairly good approximation of the ‘experimental’ response. Interestingly, the HT response in figure 9*b* is actually slightly closer to the ‘experimental’ response than is the S; HTS response. Indentation creep responses are often represented using $\log_{10}-\log_{10}$ plots so that it is worth noting that although the predicted and experimental indentation responses in figure 8*c* are indistinguishable on a $\log_{10}-\log_{10}$ scale, the uniaxial creep responses in figure 9*a* differ significantly.

For all three materials, predictions with a posterior probability $p_{1}=1.00$ have power-law creep parameters that differ from those of the corresponding ‘experimental’ material. This is because the ‘experimental’ input parameters are not in the coarse database. Even if the ‘experimental’ input parameters are in the interpolated database, interpolation errors can preclude those material parameters giving the largest value of posterior probability. For ${\mathrm{CsHSO}}_{4}$ and Sn, the predictions using both the indentation depth versus time data and the residual surface profile data (HTS) provide the best fit to the ‘experimental’ responses, while for Se (n≈1), any of the three considered datasets, HT, S and HTS, gives an identical prediction and have a posterior probability $p_{1}=1.00$.

The indentation responses for ${\mathrm{CsHSO}}_{4}$ and Sn obtained using the values of the HTS material parameters give a very good fit to the ‘experimental’ indentation responses with a posterior probability $p_{1}=1.00$ even though the predicted values of ${\dot{\epsilon }}_{0}$ are very different from the input values. This shows that for constant load and hold indentation creep, different power-law creep parameters can have very similar indentation responses.

#### (ii) Noise-contaminated data

With the noise-free ‘experimental’ responses denoted by $s^{\mathrm{input}}$ and $h^{\mathrm{input}}$, the noise-contaminated data are obtained by superposing Gaussian noise on the noise-free data by

6.5 $$s^{m}=s^{\mathrm{input}}+s^{\mathrm{noise}}\;\mathrm{and}\;h^{m}=h^{\mathrm{input}}+h^{\mathrm{noise}}.$$

The noise is added to each indentation response, $s^{\mathrm{noise}}$ and $h^{\mathrm{noise}}$, via a call to the Matlab [28] function normrnd(0,ξ,[1,K]), where 0 is the mean value, $\xi =\xi_{h}$ or $\xi_{s}$ is the standard deviation and $K=K_{h}$ or $K_{s}$ is the length of a 1×K vector of random values with the specified mean and standard deviation. Each call to normrnd provides a different vector of random values.

The standard deviation $\xi_{s}$ is related to a reference length $s_{\mathrm{ref}}$ with noise amplitude $\eta_{s}$ and the standard deviation $\xi_{h}$ is related to the maximum indentation depth with noise amplitude $\eta_{h}$ via

6.6 $$\xi_{s}=\eta_{s}s_{\mathrm{ref}}\;\mathrm{and}\;\xi_{h}=\eta_{h}\max(h_{k}^{\mathrm{input}}),$$

with $k=1,2,\ldots ,K_{h}$, $\eta_{s}\geq 0$ and $\eta_{h}\geq 0$. The reference length $s_{\mathrm{ref}}$ is taken to be the indentation depth of the noise-free surface profile after unloading.

**Figure 10.**
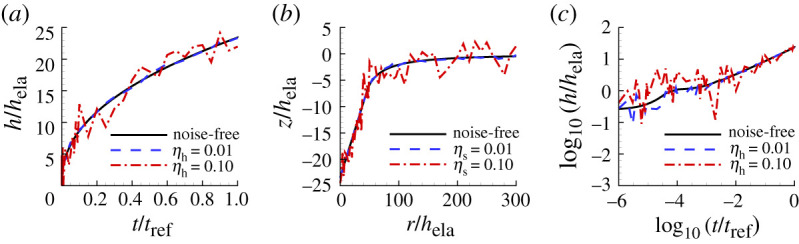
Illustration of realizations of noise-contaminated indentation data with noise amplitudes $\eta_{h}=0.01$, $\eta_{s}=0.01$, $\eta_{h}=0.10$ and $\eta_{s}=0.10$ superposed on the indentation data for Se. (*a*) Normalized indentation depth $h/h_{\mathrm{ela}}$ versus normalized time $t/t_{\mathrm{ref}}$. (*b*) Surface profiles near the indenter after unloading. (*c*) $\log_{10}$–$\log_{10}$ plot of (*a*).

Figure 10 shows examples of noise-contaminated indentation responses with values of the noise amplitudes $\eta_{h}=0.01$, $\eta_{h}=0.10$ and with $\eta_{s}=0.01$, $\eta_{s}=0.10$. The effect of noise on the prediction of the power-law creep parameters, we consider two noise levels: (i) a low noise level $\eta_{h}=\eta_{s}=0.01$; and (ii) a high noise level $\eta_{h}=\eta_{s}=0.10$.

As in [15], calculations of the posterior probability distribution are carried out for 100 realizations with the same values of the noise amplitudes $\eta_{h}$ and $\eta_{s}$. For each of the 100 realizations, the values of n, $\sigma_{0}$ and ${\dot{\epsilon }}_{0}$ having the largest posterior probability $p_{1}$ are determined. The arithmetic averages of these values are taken as the predicted power law creep parameter values associated with the specified noise amplitudes and the value of α is calculated from the resulting averaged values of n, $\sigma_{0}$ and ${\dot{\epsilon }}_{0}$. We note that no additional finite element calculations are required to determine these averaged values.

**Figure 11.**
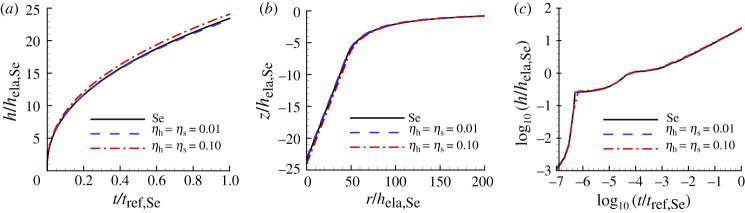
Comparison of predicted and ‘experimental’ indentation responses for Se. The associated values of n, $\sigma_{0}$, ${\dot{\epsilon }}_{0}$ and α are obtained from noise-contaminated HTS data (averaged over 100 realizations) and given in table 5. (*a*) Normalized indentation depth $h/h_{\mathrm{ela},\mathrm{Se}}$ versus normalized time $t/t_{\mathrm{ref},\mathrm{Se}}$. (*b*) Surface profiles near the indenter after unloading. (*c*) $\log_{10}$–$\log_{10}$ plot of (*a*). On the scales in this figure, the predictions with $\eta_{h}=\eta_{s}=0.01$ are indistinguishable from the corresponding ‘experimental’ responses.

Figure 11 shows the indentation responses predicted using noise-contaminated HTS data for Se compared with the corresponding noise-free ‘experimental’ responses. The responses for a low noise level ($\eta_{h}=\eta_{s}=0.01$) are indistinguishable from the experimental responses while those for a high noise level ($\eta_{h}=\eta_{s}=0.10$) still provide a good representation.

**Table 5.** Predicted values of n, $\sigma_{0}$, ${\dot{\epsilon }}_{0}$, α and the associated averaged largest posterior probability $p_{1}$ for Se obtained from averaging the predicted values over 100 realizations with $\eta_{s}=\eta_{h}=0.01$ (subscript 0.01) and with $\eta_{s}=\eta_{h}=0.10$ (subscript 0.10). See the caption of figure 6 for the meanings of HT, S and HTS.

	n	$\sigma_{0}$(MPa)	${\dot{\epsilon }}_{0}(s^{-1})$	$\alpha ({\mathrm{Pa}}^{-n}s^{-1})$	$p_{1}$
${\mathrm{HT}}_{0.01}$	1.15	5.38	$0.6\times 10^{-4}$	$1.09\times 10^{-12}$	0.31
$S_{0.01}$	1.16	8.57	$1.0\times 10^{-4}$	$0.907\times 10^{-12}$	0.78
${\mathrm{HTS}}_{0.01}$	1.16	8.67	$1.0\times 10^{-4}$	$0.895\times 10^{-12}$	0.93
${\mathrm{HT}}_{0.10}$	1.15	5.55	$0.7\times 10^{-4}$	$1.23\times 10^{-12}$	0.0066
$S_{0.10}$	1.27	6.64	$1.2\times 10^{-4}$	$0.260\times 10^{-12}$	0.0085
${\mathrm{HTS}}_{0.10}$	1.17	7.17	$0.9\times 10^{-4}$	$0.858\times 10^{-12}$	0.030

The material parameters and associated posterior probability obtained based on indentation depth versus time data (HT), residual surface profile data (S) and on both indentation depth versus time data and residual surface profile data (HTS) are given in table 5. In contrast to the noise-free case where the HT, S and HTS predictions coincided, the predictions based on different indentation data differ for noise-contaminated data. With a low noise level (subscript 0.01), the values of n and α obtained using HT data are closest to the ‘experimental’ values in table 1 even though the posterior probability value is the smallest. On the other hand, the ${\mathrm{HT}}_{0.01}$ value of ${\dot{\epsilon }}_{0}$ is 60% of the input value. The posterior probability is significantly increased when surface profile data are used in the identification analysis, increasing to $p_{1}=0.93$ for the HTS based creep parameters. The values predicted for data with a high noise level (subscript 0.10) have much larger differences from the input values and have very low values of $p_{1}$, indicating a lack of confidence in them. Although the value of $p_{1}$ for the ${\mathrm{HTS}}_{0.10}$ set of parameter values is low, it is much larger than those for the ${\mathrm{HT}}_{0.10}$ and $S_{0.10}$ predictions.

**Figure 12.**
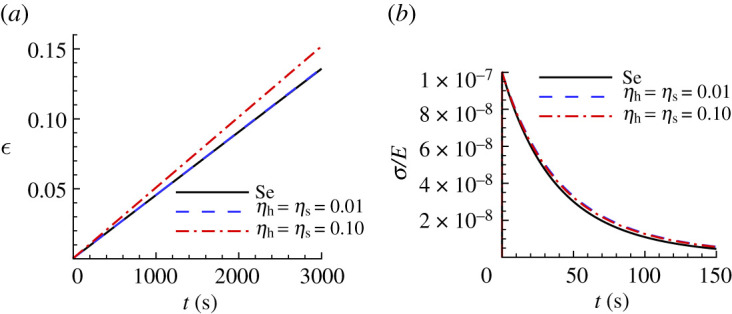
Uniaxial creep and stress relaxation responses using the averaged predicted parameter values over 100 realizations for Se obtained with noise-contaminated HTS data in table 5. The solid lines show the corresponding ‘experimental’ responses. (*a*) Uniaxial logarithmic strain, ϵ, versus time t. (*b*) Normalized uniaxial Cauchy stress, σ/E, versus time, t. On the scales in (*a*), the prediction with $\eta_{h}=\eta_{s}=0.01$ is indistinguishable from the corresponding ‘experimental’ response. In (*b*), the prediction with $\eta_{h}=\eta_{s}=0.01$ is indistinguishable from the prediction with $\eta_{h}=\eta_{s}=0.10$.

The predicted uniaxial creep and stress relaxation responses for Se obtained from one-element finite-element calculations (giving homogeneous stress and strain fields) using the creep properties in table 5 are shown in figure 12. For comparison, the corresponding responses for the ‘experimental’ material are shown. The creep parameters obtained using the low noise ${\mathrm{HTS}}_{0.01}$ indentation data provide a good fit to the uniaxial creep and stress relaxation responses. The high noise level ${\mathrm{HTS}}_{0.10}$ data also provide a rather good fit to the stress relaxation data but a much poorer fit to the uniaxial creep data. As will also be seen subsequently, the uniaxial creep response is more sensitive to the values of the creep parameters than is the stress relaxation response.

**Figure 13.**
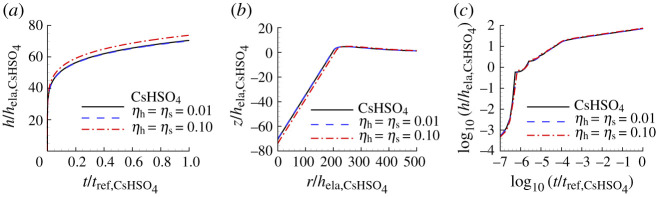
Comparison of predicted and ‘experimental’ indentation responses for ${\mathrm{CsHSO}}_{4}$. The associated values of n, $\sigma_{0}$, ${\dot{\epsilon }}_{0}$ and α are obtained from noise-contaminated HTS data (averaged over 100 realizations) and given in table 6. (*a*) Normalized indentation depth $h/h_{\mathrm{ela},{\mathrm{CsHSO}}_{4}}$ versus normalized time $t/t_{\mathrm{ref},{\mathrm{CsHSO}}_{4}}$. (*b*) Surface profiles near the indenter after unloading. (*c*) $\log_{10}$–$\log_{10}$ plot of (*a*). On the scales in (*a*) and (*b*), the predictions with $\eta_{h}=\eta_{s}=0.01$ are indistinguishable from the corresponding ‘experimental’ responses. In (*c*), all three responses are indistinguishable.

For ${\mathrm{CsHSO}}_{4}$, figure 13 compares the indentation responses predicted using noise-contaminated HTS data and the ‘experimental’ indentation responses. The responses predicted with low noise provide a very good representation of the ‘experimental’ indentation responses while the indentation depth versus time response predicted with the high noise level differs somewhat from the corresponding ‘experimental’ response.

**Table 6.** Predicted values of n, $\sigma_{0}$, ${\dot{\epsilon }}_{0}$, α and the associated averaged largest posterior probability $p_{1}$ for ${\mathrm{CsHSO}}_{4}$ obtained from averaging the predicted values over 100 realizations with $\eta_{s}=\eta_{h}=0.01$ (subscript 0.01) and with $\eta_{s}=\eta_{h}=0.10$ (subscript 0.10). See the caption of figure 6 for the meanings of HT, S and HTS.

	n	$\sigma_{0}$(MPa)	${\dot{\epsilon }}_{0}(s^{-1})$	$\alpha ({\text{Pa}}^{-n}{\text{s}}^{-1})$	$p_{1}$
${\mathrm{HT}}_{0.01}$	3.59	0.0402	51.1	$15.1\times 10^{-16}$	0.20
$S_{0.01}$	3.68	0.0502	87.3	$4.39\times 10^{-16}$	0.25
${\mathrm{HTS}}_{0.01}$	3.59	0.0551	100	$9.53\times 10^{-16}$	0.80
${\mathrm{HT}}_{0.10}$	3.60	0.0362	44.6	$17.3\times 10^{-16}$	0.0025
$S_{0.10}$	4.56	0.0316	45.5	$0.00138\times 10^{-16}$	0.0013
${\mathrm{HTS}}_{0.10}$	3.62	0.0432	63.8	$10.6\times 10^{-16}$	0.0080

The creep parameters and associated posterior probability values obtained for ${\mathrm{CsHSO}}_{4}$ from noise-contaminated data are given in table 6. The values of creep exponent n and associated pre-exponential factor α obtained based on ${\mathrm{HTS}}_{0.01}$ are in good agreement with the ‘experimental’ creep parameters in table 1 and the posterior probability is $p_{1}=0.80$. However, the value of ${\dot{\epsilon }}_{0}$, as for the prediction based on noise-free data, is 100 times that for the ‘experimental’ material. The values of α obtained using the ${\mathrm{HT}}_{0.01}$ and the $S_{0.01}$ are significantly different from the input value for ${\mathrm{CsHSO}}_{4}$ in table 1 and the posterior probability values for these predictions are much smaller than $p_{1}$ for the ${\mathrm{HTS}}_{0.01}$ prediction. The creep parameters obtained for ${\mathrm{CsHSO}}_{4}$ from the high noise level data (subscript 0.10) differ substantially from the corresponding values for the ‘experimental’ material and, consistent with this, the posterior probability values are small. Here, as in fig. 10 of [27], with increasing noise, the posterior probability distribution is more spread out with similar values of posterior probability for a range of material constitutive parameter values.

**Figure 14.**
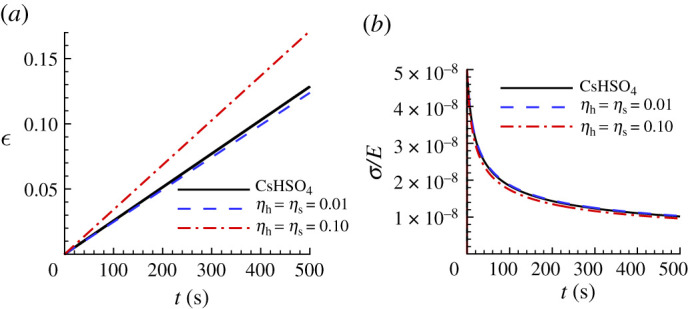
Uniaxial creep and stress relaxation responses using the averaged predicted parameter values over 100 realizations for ${\mathrm{CsHSO}}_{4}$ obtained with noise-contaminated HTS data in table 6. The solid lines show the corresponding ‘experimental’ responses. (*a*) Uniaxial logarithmic strain, ϵ, versus time t. (*b*) Normalized uniaxial Cauchy stress, σ/E, versus time, t. On the scales in (*b*), the prediction with $\eta_{h}=\eta_{s}=0.01$ is indistinguishable from the corresponding ‘experimental’ response.

Figure 14 shows a comparison between the ‘experimental’ uniaxial creep and stress relaxation responses for ${\mathrm{CsHSO}}_{4}$ and those predicted based on noise-contaminated HTS data. For both the high noise level, ${\mathrm{HTS}}_{0.10}$, based creep parameters and the low noise, ${\mathrm{HTS}}_{0.01}$, based creep parameters in table 6, there is very good agreement with the ‘experimental’ stress relaxation response in figure 14*b*. On the other hand, the creep response in figure 14*a* shows a large difference between the uniaxial creep response of the ‘experimental’ material and the prediction based on the ${\mathrm{HTS}}_{0.10}$ data.

**Figure 15.**
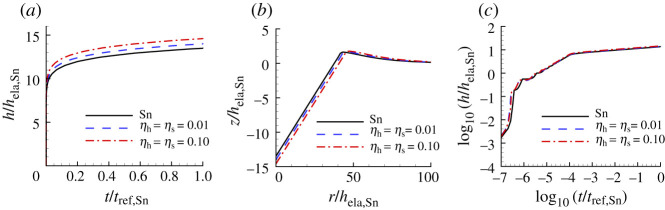
Comparison of predicted and ‘experimental’ indentation responses for Sn. The associated values of n, $\sigma_{0}$, ${\dot{\epsilon }}_{0}$ and α are obtained from noise-contaminated HTS data (averaged over 100 realizations) and given in table 7. (*a*) Normalized indentation depth $h/h_{\mathrm{ela},\mathrm{Sn}}$ versus normalized time $t/t_{\mathrm{ref},\mathrm{Sn}}$. (*b*) Surface profiles near the indenter after unloading. (*c*) $\log_{10}$–$\log_{10}$ plot of (*a*). On the scales in (*c*), all three responses are essentially indistinguishable.

The comparison of ‘experimental’ and noise-contaminated HTS data predicted indentation responses for Sn in figure 15 shows a noticeable difference even for a low noise ($\eta_{s}=\eta_{h}=0.01$) level. The HTS-based creep parameters are given in table 7 along with the associated posterior probability value. The predicted values of the pre-exponential factor α all differ substantially from the input value for Sn in table 1 except for the value based on $S_{0.01}$ and the largest value of posterior probability is only $p_{1}=0.38$ for ${\mathrm{HTS}}_{0.01}$. In contrast to the results for Se in table 5 and for ${\mathrm{CsHSO}}_{4}$ in table 6, the predicted value of α based on ${\mathrm{HTS}}_{0.01}$ data differs from the input value of Sn in table 1.

**Table 7.** Predicted values of n, $\sigma_{0}$, ${\dot{\epsilon }}_{0}$, α and the associated averaged largest posterior probability $p_{1}$ for Sn obtained from averaging the predicted values over 100 realizations with $\eta_{s}=\eta_{h}=0.01$ (subscript 0.01) and with $\eta_{s}=\eta_{h}=0.10$ (subscript 0.10). See the caption of figure 6 for the meanings of HT, S and HTS.

	n	$\sigma_{0}$(MPa)	${\dot{\epsilon }}_{0}(s^{-1})$	$\alpha ({\text{Pa}}^{-n}{\text{s}}^{-1})$	$p_{1}$
${\mathrm{HT}}_{0.01}$	6.61	12.20	24.2	$34.9\times 10^{-47}$	0.11
$S_{0.01}$	6.64	14.39	39.3	$11.6\times 10^{-47}$	0.19
${\mathrm{HTS}}_{0.01}$	6.59	15.54	47.1	$19.1\times 10^{-47}$	0.38
${\mathrm{HT}}_{0.10}$	6.54	11.92	25.5	$134\times 10^{-47}$	0.0016
$S_{0.10}$	6.25	15.50	40.4	$4643\times 10^{-47}$	0.0013
${\mathrm{HTS}}_{0.10}$	6.63	13.43	33.1	$18.2\times 10^{-47}$	0.0048

**Figure 16.**
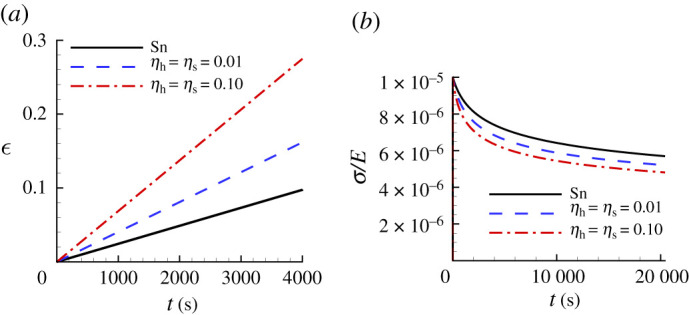
Uniaxial creep and stress relaxation responses using the averaged predicted parameter values over 100 realizations for Sn obtained with noise-contaminated HTS data in table 7. The solid lines show the corresponding ‘experimental’ responses. (*a*) Uniaxial logarithmic strain, ϵ, versus time t. (*b*) Normalized uniaxial Cauchy stress, σ/E, versus time, t.

The noise-contaminated uniaxial creep and stress relaxation predictions for Sn in figure 16 show a significant deviation from the corresponding responses of the ‘experimental’ material. In particular, in figure 16*a*, the creep responses predicted based on both the ${\mathrm{HTS}}_{0.01}$ data and the ${\mathrm{HTS}}_{0.10}$ data are very different from the responses of the ‘experimental’ material.

For all three materials, values of n and α were calculated using a different 100 realizations. The ${\mathrm{HTS}}_{0.01}$ predicted values of n were the same to three significant figures and the values of α differed by 1 in the third significant figure.

The results here show an increasing sensitivity to noise with increasing creep stress exponent n, with relatively little sensitivity for Se (n=1.15), more sensitivity for ${\mathrm{CsHSO}}_{4}$ (n=3.59) and the most noise sensitivity for Sn (n=6.60).

### (b) Comparison with analytical models

The aim of the analytical power-law creep models is to provide explicit expressions for relating measured indentation responses to the constitutive parameters n and α in equation (2.6). The first step is to identify the power-law creep regime of the $h/h_{\mathrm{ela}}$ versus time t responses. The steady-state portions of the indentation depth, $h/h_{\mathrm{ela}}$, versus time, t, responses in figure 2*c* are taken to be $-2\leq \log_{10}(t/t_{\mathrm{ref}})\leq 0$ for Se; $-3\leq \log_{10}(t/t_{\mathrm{ref}})\leq 0$ for ${\mathrm{CsHSO}}_{4}$ and Sn. A least-squares fit is used and, based on equation (5.6), the slope of the $\log_{10}-\log_{10}$ plot is identified with 1/(2n) and β is obtained from the least-squares intercept. The least-square fit used to determine the value of n involved 197, 455 and 253 points for Se, ${\mathrm{CsHSO}}_{4}$ and Sn, respectively. The values of n and β so obtained are then used in analytical expressions for power-law creep indentation derived by Bower *et al.* [1] and Ginder *et al.* [2] to obtain the value of the pre-exponential factor α.

Using expressions derived by Bower *et al.* [1] and identifying p in equation (5.2) with the force per unit nominal area as in Su *et al.* [10]:

6.7 $$\alpha_{\mathrm{BFNO}}=\beta (F^{n}c^{2n-1})\cot\gamma ,$$

where both F and c are functions of n and the indenter angle γ (figure 1). The values of F and c were estimated using the values for an indenter angle $\gamma =70^{\circ }$ in tables 1 and 2 of [10].

The closed-form algebraic expression for α obtained by Ginder *et al.* [2] based on an expanding cavity model is

6.8 $$\alpha_{\mathrm{GNP}}=\beta (\frac{2n}{3}{)}^{n}\cot\gamma .$$

For the noise-contaminated predictions of the analytical models, noise is added to the power-law regime indentation depth versus time data using the Matlab [28] function normrnd(0,ξ,[1,K]) where the K is the number of data points on the indentation depth versus time response that lie in the power-law regime (i.e. 197–455 points). Note that although the mean and standard deviation are the same as for the Bayesian-based calculations in §6a(ii), the number of data points and the specific realizations differ. The values of n and α for noise-contaminated data were again obtained by averaging over 100 realizations. As for the Bayesian statistics-based predictions, carrying out the noise-contaminated calculations for a different 100 realizations with $\eta_{h}=\eta_{s}=0.01$ does not significantly change the results. Table 8 shows a comparison between the values of the creep exponent n and the pre-exponential factor α associated with the input experimental materials, the Bayesian statistical analysis, the expression equation (6.7) and the expression equation (6.8). Note that the Bayesian statistics based predictions shown are based on HTS data whereas the analytical model predictions only use HT data. Also, because the values of n used in equations (6.7) and (6.8) are obtained from the least-squares fits to computed power-law regime responses, the same value of n appears for the BFNO and GNP entries in table 8.

**Table 8.** A comparison of the values of n, α and $\alpha \sigma_{a}^{n}$ obtained using the input ‘experimental’ data in table 1 (subscript inp), the Bayesian-type statistical approach with HTS data from §6a(i)(ii) (subscript Bayes), HT data with equation (6.7) (subscript BFNO), and HT data with equation (6.8) (subscript GNP). The subscript ${\left(\;\right)}_{\mathrm{nf}}$ denotes noise-free data and the subscript ${\left(\;\right)}_{0.01}$ denotes values averaged of predictions over 100 realizations with $\eta_{h}=\eta_{s}=0.01$. Also shown is the value of $\alpha \sigma_{a}^{n}$ where for each material, $\sigma_{a}$ is the applied stress in equation (6.1). The values of n used in equations (6.7) and (6.8) were obtained from a least-squares fit to the steady-state portions of the ‘experimental’ indentation depth versus time responses.

	Se	${\mathrm{CsHSO}}_{4}$	Sn
$n_{\mathrm{inp}}$	1.15	3.59	6.60
$\alpha_{\mathrm{inp}}\;({\mathrm{Pa}}^{-n}\;s^{-1})$	$1.04\times 10^{-12}$	$9.89\times 10^{-16}$	$9.97\times 10^{-47}$
$\alpha_{\mathrm{inp}}\sigma_{a}^{n}\;(s^{-1})$	$4.50\times 10^{-5}$	$2.57\times 10^{-4}$	$2.44\times 10^{-5}$
$n_{\mathrm{Bayes},\mathrm{nf}}$	1.16	3.58	6.46
$\alpha_{\mathrm{Bayes},\mathrm{nf}}\;({\mathrm{Pa}}^{-n}\;s^{-1})$	$0.898\times 10^{-12}$	$10.6\times 10^{-16}$	$86.7\times 10^{-47}$
$\alpha_{\mathrm{Bayes},\mathrm{nf}}\sigma_{a}^{n}\;(s^{-1})$	$4.53\times 10^{-5}$	$2.56\times 10^{-4}$	$2.81\times 10^{-5}$
$n_{\mathrm{Bayes},0.01}$	1.16	3.59	6.59
$\alpha_{\mathrm{Bayes},0.01}\;({\mathrm{Pa}}^{-n}\;s^{-1})$	$0.895\times 10^{-12}$	$9.53\times 10^{-16}$	$19.1\times 10^{-47}$
$\alpha_{\mathrm{Bayes},0.01}\sigma_{a}^{n}\;(s^{-1})$	$4.52\times 10^{-5}$	$2.48\times 10^{-4}$	$4.04\times 10^{-5}$
$n_{\mathrm{BFNO},\mathrm{nf}}$	1.17	3.57	6.66
$\alpha_{\mathrm{BFNO},\mathrm{nf}}\;({\mathrm{Pa}}^{-n}\;s^{-1})$	$0.813\times 10^{-12}$	$11.6\times 10^{-16}$	$3.91\times 10^{-47}$
$\alpha_{\mathrm{BFNO},\mathrm{nf}}\sigma_{a}^{n}\;(s^{-1})$	$4.78\times 10^{-5}$	$2.60\times 10^{-4}$	$2.27\times 10^{-5}$
$n_{\mathrm{BFNO},0.01}$	1.17	3.57	6.66
$\alpha_{\mathrm{BFNO},0.01}\;({\mathrm{Pa}}^{-n}\;s^{-1})$	$0.832\times 10^{-12}$	$11.8\times 10^{-16}$	$4.59\times 10^{-47}$
$\alpha_{\mathrm{BFNO},0.01}\sigma_{a}^{n}\;(s^{-1})$	$4.89\times 10^{-5}$	$2.65\times 10^{-4}$	$2.67\times 10^{-5}$
$n_{\mathrm{GNP},\mathrm{nf}}$	1.17	3.57	6.66
$\alpha_{\mathrm{GNP},\mathrm{nf}}\;({\mathrm{Pa}}^{-n}\;s^{-1})$	$0.769\times 10^{-12}$	$5.09\times 10^{-16}$	$12.3\times 10^{-47}$
$\alpha_{\mathrm{GNP},\mathrm{nf}}\sigma_{a}^{n}\;(s^{-1})$	$4.52\times 10^{-5}$	$1.14\times 10^{-4}$	$7.15\times 10^{-5}$
$n_{\mathrm{GNP},0.01}$	1.17	3.57	6.66
$\alpha_{\mathrm{GNP},0.01}\;({\mathrm{Pa}}^{-n}\;s^{-1})$	$0.790\times 10^{-12}$	$5.18\times 10^{-16}$	$14.2\times 10^{-47}$
$\alpha_{\mathrm{GNP},0.01}\sigma_{a}^{n}\;(s^{-1})$	$4.65\times 10^{-5}$	$1.16\times 10^{-4}$	$8.25\times 10^{-5}$

The values of α for the various entries in table 8 are not directly comparable since the units of α vary with n. However, the quantity $\alpha \sigma_{a}^{n}$ has the dimension 1/time and can be directly compared. In the power-law creep regime, the uniaxial creep strain rate in equation (6.2) is given by $\alpha \sigma_{a}^{n}$, with $\sigma_{a}$ the applied stress. Thus, the comparison between the various predictions for $\alpha \sigma_{a}^{n}$ with the ‘experimental’ value provides a measure of the accuracy of the prediction.

For Se (n=1.15), all the predictions of n and $\alpha \sigma_{a}^{n}$, both for noise-free data and for noise-contaminated data (with $\eta_{h}=\eta_{s}=0.01$ in table 8) provide a good representation of the ‘experimental’ material. Perhaps surprisingly, the simple formula in equation (6.8) provides a slightly more accurate prediction than equation (6.7).

For ${\mathrm{CsHSO}}_{4}$ (n=3.59), the ‘experimental’ values of n and $\alpha \sigma_{a}^{n}$ are well represented by the Bayesian statistical predictions and by equation (6.7) while the predictions of $\alpha \sigma_{a}^{n}$ from equation (6.8) differ from the ‘experimental’ value by a factor of about 2.

For Sn (n=6.60), the Bayesian statistical prediction and the prediction based on equation (6.7) are both rather accurate for noise-free data. The prediction based on equation (6.7) also provides a reasonably accurate value of $\alpha \sigma_{a}^{n}$ for the noise-contaminated data while the Bayesian statistics based prediction of $\alpha \sigma_{a}^{n}$ differs from the ‘experimental’ value. This may be due to the values of n and β used in equation (6.7) being obtained directly from the power-law regime indentation data, whereas the Bayesian statistics values of n and α are obtained based on database data which largely consist of interpolated approximations. Nevertheless, the Bayesian statistics values of n and α based on noisy data are rather close to the ‘experimental’ input values of Sn.

The accuracy of the predictions becomes more sensitive to noise for larger values of the stress exponent n. For example, for Se (n=1.15) with $\eta_{h}=\eta_{s}=0.10$, the predicted values of $\alpha \sigma_{a}^{n}=5.04\times 10^{-5}\;s^{-1}$, $6.23\times 10^{-5}\;s^{-1}$, and $7.40\times 10^{-5}\;s^{-1}$ for the Bayesian statistics approach, equation (6.7) and equation (6.8), respectively. For Sn (n=6.60), the corresponding values are $6.86\times 10^{-5}\;s^{-1}$, $1.61\times 10^{-2}\;s^{-1}$ and $2.89\times 10^{-2}\;s^{-1}$. Hence, for very noisy data, both analytical approximations for Sn (n=6.60) are very inaccurate.

## 7. Conclusion

The Bayesian-type statistical approach of Zhang *et al.* [15] has been used to identify the power-law creep constitutive parameters, the creep exponent n and the pre-exponential factor α, from ‘experimental’ load and hold indentation creep measurements, considering noise-free as well as noise-contaminated data. The indentation creep measurements are: (i) the indentation depth versus time response and (ii) the residual surface profile. Material properties representative of three materials have been considered: amorphous selenium (Se), solid acid ${\mathrm{CsHSO}}_{4}$ and tin (Sn). Finite-element calculations were carried out to populate a coarse database of power-law creep parameters. The finer database used for the Bayesian statistical analyses was created by interpolation. Uniaxial creep and stress relaxation responses were computed using the power-law creep parameters obtained from the Bayesian-type statistical approach using noise-free as well as noise-contaminated data and compared with the corresponding responses of the ‘experimental’ materials. The Bayesian statistics-based predictions were also compared with predictions based on analytical power-law creep indentation expressions of Bower *et al.* [1] and Ginder *et al.* [2].

1. The Bayesian-type statistical approach provides the values of power-law creep parameters that provide a good fit to the indentation responses of all the materials considered when based on noise-free data and for sufficiently small noise amplitudes. The sensitivity to noise increases with increasing creep stress exponent n.
  — For Se (n=1.15), the creep parameters obtained from both the noise-free and noise-contaminated indentation responses provide a good fit to the uniaxial creep and stress relaxation responses.
  — For Sn (n=6.60), creep parameters that provide good fit to the load and hold indentation responses do not necessarily give a good fit to the uniaxial creep and stress relaxation responses.
2. Can very different power-law creep parameters give nearly the same responses in load and hold indentation creep? In the circumstances analysed, different values of the power law creep parameters did give reasonably good fits to the ‘experimental’ indentation data, particularly for noisy data, but no cases were found where very different values of both power-law creep parameters gave nearly the same indentation response.
3. Does using the residual surface profile in addition to or instead of the indentation depth versus time data improve the quality of the prediction? Using both indentation depth versus time data and residual surface profile data generally leads to an improved prediction of the uniaxial creep and stress relaxation responses. For Se (n=1.15), the improvement over only using indentation depth versus time data is negligible.
4. How sensitive is the predicted creep response to noise in the ‘experimental’ indentation data? The uniaxial creep response is more sensitive to the accuracy of the predicted values of the power-law creep parameters, and therefore to noise, than is the uniaxial stress relaxation response.
5. How do the power-law creep properties obtained using the analytical steady-state creep results of Bower *et al.* [1] and Ginder *et al.* [2] compare with those predicted from the Bayesian-type statistical approach? For Se (n=1.15), the predictions of both the analytical models of Bower *et al.* [1] and of Ginder *et al.* [2] are in very good agreement with those of the ‘experimental’ material, while the model of Bower *et al.* [1] provides a good fit for all three values of creep stress exponent and the corresponding pre-exponential factor considered if the noise level is sufficiently small.

## Data accessibility

The article data are available from the Dryad Digital Repository: https://doi.org/10.5061/dryad.1zcrjdfrg [29].

## Appendix A. Values of ζ for constant load and hold indentation creep

The tabulated values of $\zeta (n,{\dot{\epsilon }}_{0}t_{2})$ are given in equations (A1) to (A3). For the values of n and ${\dot{\epsilon }}_{0}t_{2}$ that are not tabulated, the value of $\zeta (n,{\dot{\epsilon }}_{0}t_{2})$ used in equation (5.1) is obtained by linear interpolation between tabulated values.

A 1 $$\zeta (1,{\dot{\epsilon }}_{0}t_{2})=\left\{ \begin{array}{ll} 4.7\times 10^{4}, & {\dot{\epsilon }}_{0}t_{2}=0.1\\ 4.7\times 10^{3}, & {\dot{\epsilon }}_{0}t_{2}=1.0\\ 4.7\times 10^{2}, & {\dot{\epsilon }}_{0}t_{2}=10.0\\ 4.7\times 10^{1}, & {\dot{\epsilon }}_{0}t_{2}=100.0 \end{array}\right.\;\zeta (2,{\dot{\epsilon }}_{0}t_{2})=\left\{ \begin{array}{ll} 2.4\times 10^{5}, & {\dot{\epsilon }}_{0}t_{2}=0.1\\ 4.7\times 10^{4}, & {\dot{\epsilon }}_{0}t_{2}=1.0\\ 1.4\times 10^{4}, & {\dot{\epsilon }}_{0}t_{2}=10.0\\ 4.7\times 10^{3}, & {\dot{\epsilon }}_{0}t_{2}=100.0 \end{array}\right.$$

A 2 $$\zeta (3,{\dot{\epsilon }}_{0}t_{2})=\left\{ \begin{array}{ll} 9.4\times 10^{4}, & {\dot{\epsilon }}_{0}t_{2}=0.1\\ 4.7\times 10^{4}, & {\dot{\epsilon }}_{0}t_{2}=1.0\\ 1.9\times 10^{4}, & {\dot{\epsilon }}_{0}t_{2}=10.0\\ 9.4\times 10^{3}, & {\dot{\epsilon }}_{0}t_{2}=100.0 \end{array}\right.\;\zeta (4,{\dot{\epsilon }}_{0}t_{2})=\left\{ \begin{array}{ll} 1.4\times 10^{5}, & {\dot{\epsilon }}_{0}t_{2}=0.1\\ 1.1\times 10^{5}, & {\dot{\epsilon }}_{0}t_{2}=1.0\\ 9.4\times 10^{4}, & {\dot{\epsilon }}_{0}t_{2}=10.0\\ 3.8\times 10^{4}, & {\dot{\epsilon }}_{0}t_{2}=100.0 \end{array}\right.$$

A 3 $$\zeta (5,{\dot{\epsilon }}_{0}t_{2})=\left\{ \begin{array}{ll} 1.4\times 10^{5}, & {\dot{\epsilon }}_{0}t_{2}=0.1\\ 1.1\times 10^{5}, & {\dot{\epsilon }}_{0}t_{2}=1.0\\ 9.4\times 10^{4}, & {\dot{\epsilon }}_{0}t_{2}=10.0\\ 7.5\times 10^{4}, & {\dot{\epsilon }}_{0}t_{2}=100.0 \end{array}\right.\;\zeta (n>5,{\dot{\epsilon }}_{0}t_{2})=\left\{ \begin{array}{ll} 1.4\times 10^{5}, & {\dot{\epsilon }}_{0}t_{2}=0.1\\ 1.1\times 10^{5}, & {\dot{\epsilon }}_{0}t_{2}=1.0\\ 9.4\times 10^{4}, & {\dot{\epsilon }}_{0}t_{2}=10.0\\ 9.4\times 10^{4}, & {\dot{\epsilon }}_{0}t_{2}=100.0 \end{array}\right.$$
